# Emergency medical services: the next linking asset for public health approaches to palliative care?

**DOI:** 10.1177/26323524231163195

**Published:** 2023-04-13

**Authors:** Madeleine L. Juhrmann, Andrea E. Grindrod, Caleb H. Gage

**Affiliations:** Northern Clinical School, Faculty of Medicine and Health, University of Sydney, Sydney, NSW, Australia; The Palliative Centre, Greenwich Hospital, HammondCare, Greenwich, NSW, Australia; Public Health Palliative Care Unit, School of Psychology and Public Health, La Trobe University, Melbourne, VIC, Australia; Division of Emergency Medicine, University of Cape Town, Cape Town, South Africa

**Keywords:** ambulance, emergency medical service, palliative care, paramedic, public health, terminal care

## Abstract

Emergency medical services (EMS) are a unique workforce providing 24/7 emergency care across high-income countries (HICs) and low- and middle-income countries (LMICs). Although traditionally perceived as first responders to traumatic and medical emergencies, EMS scope of practice has evolved to respond to the changing needs of communities, including a growing demand for community-based palliative care. Public health provides a useful framework to conceptualise palliative and end-of-life care in community-based settings. However, countries lack public policy frameworks recognising the role EMS can play in initiating palliative approaches in the community, facilitating goals of care at end of life and transporting patients to preferred care settings. This article aims to explore the potential role of EMS in a public health palliative care approach in a critical discussion essay format by (1) discussing the utility of EMS within a public health palliative care approach, (2) identifying the current barriers preventing public health approaches to EMS palliative care provision and (3) outlining a way forward through priorities for future research, policy, education and practice. EMS facilitate equitable access, early provision, expert care and efficacious integration of community-based palliative care. However, numerous structural, cultural and practice barriers exist, appearing ubiquitous across both HICs and LMICs. A Public Health Palliative Care approach to EMS Framework highlights the opportunity for EMS to work as a linking asset to build capacity and capability to support palliative care in place; connect patients to health and community supports; integrate alternative pathways by engaging multidisciplinary teams of care; and reduce avoidable hospital admissions by facilitating home-based deaths. This article articulates a public health approach to EMS palliative and end-of-life care provision and offers a preliminary framework to illustrate the components of a potential implementation and policy strategy.

## Introduction

Emergency medical services (EMS) are specifically staffed, designed and equipped for the emergency care of patients across metropolitan, regional, rural and remote settings 24 h a day, 7 days a week (24/7). EMS are traditionally perceived as first responders to traumatic and medical emergencies, typically motor vehicle accidents or cardiac arrests.^1^ However, as a result of global populations living longer and with more chronic progressive diseases, the EMS scope of practice has evolved to respond to these changing needs of communities.^2^ Ageing populations and increasing preferences to die at home have resulted in a growing demand for community-based palliative care.^3^

The World Health Organization (WHO) defines palliative care as

An approach that improves the quality of life for patients and their families who are facing problems associated with life-limiting illnesses, through the prevention and relief of suffering by means of early identification, correct assessment and treatment of pain and other problems, whether physical, psychosocial or spiritual.^4^

End-of-life care (EoLC) typically refers to assessment, care and treatment of patients in their final 12 months prior to death.^5^

Specialist palliative care services are not able to fulfil the needs of these patients in isolation, particularly after hours.^6^ As a result, EMS have emerged as ‘gap-fillers’, providing palliative and EoLC to patients in community-based settings when other services are unavailable, as palliative emergencies arise.^1^ This shifting practice of EMS is apparent across both high-income countries (HIC) and low- and middle-income countries (LMIC).^7–12^

Public health provides a useful framework to conceptualise palliative and EoLC in community-based settings,^13–15^ recognising that health is determined by a range of social and structural factors impacting on equity of access to palliative care,^16,17^ while highlighting the intersecting role of health services and citizens in delivering holistic EoLC, and recognising everyone has a role to play in EoLC.^18^

Palliative care networks have made significant progress to integrate multidisciplinary teams into their provision of care, including emergency departments (EDs).^19,20^ However, internationally, countries lack public policy frameworks that acknowledge the unique role EMS can play in initiating palliative approaches in the community, facilitating goals of care at end of life and transporting patients to preferred care settings.^21^ Furthermore, the WHO neglects to recognise the role of EMS in key policy documents despite highlighting ‘palliative care requires a broad multidisciplinary approach’,^22^ in addition to ‘multisectoral policy and action to address broader determinants of health’.^23^

Current attempts to integrate EMS with palliative care are predominately focussed on protocols and training.^24,25^ However, in this discussion essay, we will argue for a broader approach – at a national and global level – which implements integrative policy to compliment education and clinical practice guidelines. To address these gaps, this article aims to explore the potential role of EMS in a public health palliative care approach by (1) discussing the utility of EMS within a public health palliative care approach, (2) identifying the current barriers preventing public health approaches to EMS palliative care provision and (3) outlining a way forward through priorities for future research, policy, education and practice.

## Utility of EMS in a public health palliative care approach

EMS are uniquely positioned in healthcare systems as they provide a link between community-based patients and in-hospital settings. This position can be leveraged in the provision of palliative care through EMS facilitating equitable access, early provision, expert care and efficacious integration.

EMS provide access to healthcare through their mobility and round the clock availability. In addition to the provision of traditional emergency care, EMS can deliver palliative care to patients in community-based settings and transport those requiring further specialist care to alternative destinations beyond the ED, including in-patient palliative care units.^26^ EMS also offer significant adjunct in-home support to community-based palliative care patients during out-of-hours times when other palliative care services are often unavailable.^27,28^

Internationally, people of low socioeconomic status (SES) and those marginalised from mainstream society are particularly susceptible to experience barriers in receiving palliative care in keeping with the disproportionate challenges they face accessing services in general. Such marginalised and underserved groups may include, but are not limited to, people who are homeless or at risk of homelessness;^29^ living in regional, rural and remote areas;^30^ living with disability;^31^ LGBTQI+ identifying;^32^ First Nations identifying;^33^ culturally and linguistically diverse;^34^ and incarcerated.^35^ Targeted initiatives to broaden access to such groups to address these inequities could be facilitated through the provision of EMS palliative care reaching diverse global communities. These groups are often picked up in crisis situations as a direct result of inequitable access.^36,37^

EMS may be the first point of medical contact for palliative care patients,^38^ and are therefore well-positioned to initiate early palliative care at home.^39^ This potential benefit for patient comfort, early identification and relief of suffering is significant.^11^ As many palliative patients wish to receive care in their homes and avoid transportation to hospitals, with appropriate clinical support, EMS can provide community-based palliative care that ensures patient wishes and autonomy are respected.^40^

The early and timely provision of community-based palliative care has been shown to positively impact both patients and their conditions, improving patient and family quality of life (QoL) satisfaction and confidence.^41^ A recent study implementing a specialist EMS palliative care programme resulted in high patient and family satisfaction, and greater consumer recognition of the compassion and skill of EMS personnel when providing palliative symptom management. In addition, the knowledge of the 24/7 availability of the EMS providers in this programme provided confidence and peace of mind for patients, their families and carers, facilitating early grief support and reducing avoidable hospital admissions.^26^

As palliative care patients advance in age, EoLC emergencies are more likely, for which EMS are often called.^42,43^ Alarming conditions such as acute pain, dyspnoea, convulsions and sudden loss of consciousness are well documented reasons for EMS attendance to palliative patients.^1,44,45^ These conditions, *inter alia*, may cause significant distress for patients, their families and caregivers;^44^ however, they also represent areas of EMS expertise which can be readily resolved. As out-of-hospital specialists, EMS are well trained and experienced in managing these emergencies in community-based settings.^11^ As such, EMS are well-equipped to attend to palliative care patients in the likely event they deteriorate and require immediate care, especially when only generalist palliative care capacity is required out-of-hours.

Palliative care is, by its very nature, multidisciplinary.^41^ However, the EMS discipline represents a field which is rarely involved in formal palliative care partnerships, despite regularly interacting with these patients.^40,46,47^ Integrating EMS with other palliative care service providers offers a range of compelling opportunities to enhance public health responses to palliative care. EMS could initiate palliative care to unidentified patients who would benefit from this approach to care. In doing so, EMS personnel are in a unique position to gather first-hand insight into patients’ social and structural determinants of health, which may inform future palliative care planning and management. EMS could also connect patients, their families and carers with other relevant community services, including social workers and bereavement groups, thereby activating integrated healthcare pathways.^38^ In this way, EMS has the opportunity to provide efficacious integration through a ‘treat and refer’ function.^48^

Based on these unique abilities of EMS and the resulting benefits to palliative care provision, a public health approach to EMS palliative care is warranted. Targeted public health policy may assist in the integration of EMS with other healthcare systems working within palliative care service provision. Interdisciplinary partnerships could facilitate the delivery of early and home-based palliative care, respect of patient autonomy, improved patient and family QoL, increased patient and family satisfaction and confidence, decreased healthcare costs and enhanced fulfilment of community preferences to die at home.^26^

LMICs may particularly benefit from strengthening public health policy relating to EMS and palliative care system integration. The potential decreased healthcare costs conferred by integrating already existing systems (EMS and palliative care) would represent an efficient use of scarce resources, especially when avoidable hospital admissions are significantly reduced in preference for community-based care.

## Barriers preventing public health palliative care approaches to EMS

Despite the aforementioned utility of integrating EMS and palliative care systems through a public health policy approach, several common barriers exist that often result in lamentable patient care,^49^ as illustrated by the case study vignette. These barriers, which appear ubiquitous across both HICs and LMICs,^11,45^ may be divided into three categories: structural, cultural and practice. We argue changes to public health policy could assist in overcoming many of these barriers.

EMS systems are traditionally designed and perceived by consumers to provide emergency treatment and patient conveyance to tertiary facilities.^1^ However, for patients at end of life, transportation is often undesired and inappropriate.^50^ Despite patient and family wishes often contradicting this trajectory, EMS regularly convey palliative patients to a medical facility,^50^ and due to a lack of alternate pathways, this almost invariably involves an ED. Murphy-Jones and Timmons described patient transport as a default ‘safety net’ for EMS providers when managing palliative patients.^50^ Where EMS providers deviate from standard practice, there is a perceived lack of legal support and fear of litigation.^51^

**Table:** 

Case study vignette EMS are dispatched to a cancer patient with dyspnoea. Upon arrival, an 88-year-old male with extensive stage, small cell, lung carcinoma presents. His level of consciousness is decreased, and severe respiratory distress is evident. Attendant adult children of the patient called EMS as they were startled by their father’s suffering and were unsure how to relieve his troubled breathing. They express knowledge that their father is dying but wishes to remain at home with his family. In the absence of legal documentation, the EMS crew contact their clinical advisor who recommends treatment and transport to hospital for definitive care. The EMS crew follow this advice, intubate the patient and initiate mechanical ventilation. During transport the patient becomes haemodynamically unstable. An adrenaline infusion is initiated to “keep the patient going” until hospital arrival. “No one dies in the back of the ambulance” being the (un)spoken rule. Shortly after hospital handover, the patient arrests and emergency department staff withhold resuscitative efforts due to the nature of the patient and the now present Do Not Resuscitate (DNR) order. Discussing the case, the EMS crew are satisfied they performed to the best of their abilities and “at least managed to get the patient to hospital alive”.

EMS, emergency medical services.

However, family members may also call EMS during a palliative care emergency to prevent the dying process of their loved one, and advocate for a patient to be taken to the hospital even if preferences and plans to die at home have been documented.^52^ Structural and individual inequities also influence a palliative care patient and their family and carers’ capacity to facilitate a home-based death.^53^ Literature suggests greater hospital use in the last year of life of patients living in more disadvantaged socioeconomic positions.^54^ As a result, EMS ought to be cognisant of these disparities and have the ability to make holistic assessments when considering the need to transport a palliative care patient to hospital.

Medico-legal documentation represents a further challenge to EMS provider decision-making in palliative situations. Advance directives (AD) and do not resuscitate (DNR) orders, for example, are designed to assist decision-making in these scenarios. However, they are largely unavailable.^38,55^ Despite this intent, where these documents do exist, they tend to cause greater confusion for EMS providers as there are no clear guidelines for their use and the validity of such documentation in emergency scenarios is questioned.^12,56^

EMS system rigidity and the fear of medico-legal repercussions act as significant structural barriers preventing EMS personnel from challenging the traditional expectations of hospital-based care, and pursuing alternative pathways.^57^

The typical focus of EMS involves immediate measures to preserve life or limb until definitive care is reached.^51^ EMS providers are trained to intervene, often invasively, in life-threatening situations and convey their patients to a medical facility for definitive care.^48^ In this manner, EMS providers are predisposed to perform curative interventions with the aim of ‘saving lives’.^51,55^ As a result, the EMS culture is death averse, clearly distinguished from the more holistic palliative approach primarily concerned with the prevention and relief of suffering.^4^ Palliative therapeutic goals may, therefore, come into conflict with EMS therapeutic goals.^49,51,58^ Thus, when EMS providers are confronted with palliative patients, their interventions may be inappropriate and even harmful.

Because of these disparities, some EMS systems and individual EMS personnel may not identify palliative care as part of their role. To combat this cultural barrier, Lamba *et al.*^38^ recommended first identifying ‘EMS-palliative care champions’ within services who have a strong affinity and willingness to build capacity within services. Despite possible heterogeneity of EMS views and opinions, EMS systems have played increasingly prominent roles in the provision of community-based care.^39^ Within this model, there has been a greater recognition of the capacity and capability of EMS to support palliative care in place, and facilitate alternative referral pathways to other health providers when required.^26,39,46,49^

Structural and cultural barriers facing EMS and palliative care system integration directly impact practice. In the case study vignette, the structural barriers of compulsory patient transport and lack of legal documentation, alongside the cultural barrier of death adversity among EMS personnel, resulted in poor practice by the EMS system. Futile interventions were performed, and patient autonomy was ignored. To overcome these barriers, EMS could have access to a 24/7 palliative care specialist via telehealth, who could reassure the EMS personnel to take a palliative approach to care and provide the necessary medico-legal documentation to verify this course of action. As a result, the EMS personnel could administer an opioid to relieve the dyspnoea, provide reassurance to the patient and their family, and leave them at home with follow-up referral to a community palliative care team during regular hours. As a result of this change in practice, a desirable outcome for the patient, their family and the broader healthcare system could be achieved.

While structural and cultural barriers must be overcome to improve practice, unique barriers remain regarding the availability of resources, EMS scope of practice and complex nature of the community-based setting.^11^ While several HICs with sufficient resources have developed specialist EMS personnel roles integrating the provision of palliative care (i.e. Extended Care Paramedics in Australia^59^ and Community Paramedics in Canada),^60^ LMICs lack the necessary resources to develop these novel roles and require individualised approaches given their disparate contexts. Furthermore, not all EMS personnel fall within the same scope of practice. Those with generalist training may be constrained in their ability to practice palliative care, such as a limited scope to administer opioids, especially through the preferred subcutaneous route for palliative patients.^61^ The out-of-hospital setting may further challenge EMS’ ability to practice palliative care given the uncontrolled and unpredictable environment, often resulting in a dearth of information available to EMS personnel.^38,40,46^

## A way forward

Despite the advances made to connect multidisciplinary teams across palliative care settings, too often the potential for EMS integration is missed. Throughout this discourse, a prominent question has been raised: how can EMS better engage with community networks and health/social services to deliver improved outcomes for palliative care patients, families and carers, and empower EMS personnel to confidently adopt palliative approaches to care?

In the past, mental health emergencies have also been perceived as beyond the traditional scope of practice for EMS.^62^ However, as a result of increasingly prevalent mental health emergencies in HIC communities,^63,64^ coupled with the de-institutionalisation of mental healthcare leading to a greater focus on person-centred community-based care, EMS and other first responder services have adapted their attitudes and approach. Integrated models of mental health emergency first responder care are emerging across HICs, which place mental health workers in police or ambulance call centres, or co-response mobile crisis services alongside police officers and EMS personnel.^65^ Specialist mental health workers can provide expert assessment to patients at point of care, reducing the need to invoke involuntary detention on the patient and avoidable transportation to hospital for further assessment.^66^

Police, ambulance, clinician early response (PACER) is an Australian tri-response mobile service which teams a mental health clinician, police officer and EMS personnel together in a first responder vehicle to attend mental health crisis in the community.^67^ The pilot programme in one Australian jurisdiction saw 90% of patients assessed by the PACER team able to stay in the community, with only 12% of people requiring transport to the ED – a significant decrease from 56% previously.^68^

Given integrated models of care can have a significant impact on improving timeliness of care pathways and diversions from EDs, a palliative care co-response mobile service could potentially better facilitate community preferences to die at home and engender stronger public health approaches to palliative care, if partnerships with community networks were also pursued. Literature supports the notion that care begins when the emergency number is dialled, highlighting opportunities for palliative care specialists to also be integrated into EMS call centres.^69^ However, limitations to both models must be recognised, especially in LMICs and regional, rural and remote areas of HICs, where resources can be scarce.

## Priorities for research, education, policy and practice

**Figure 1.**
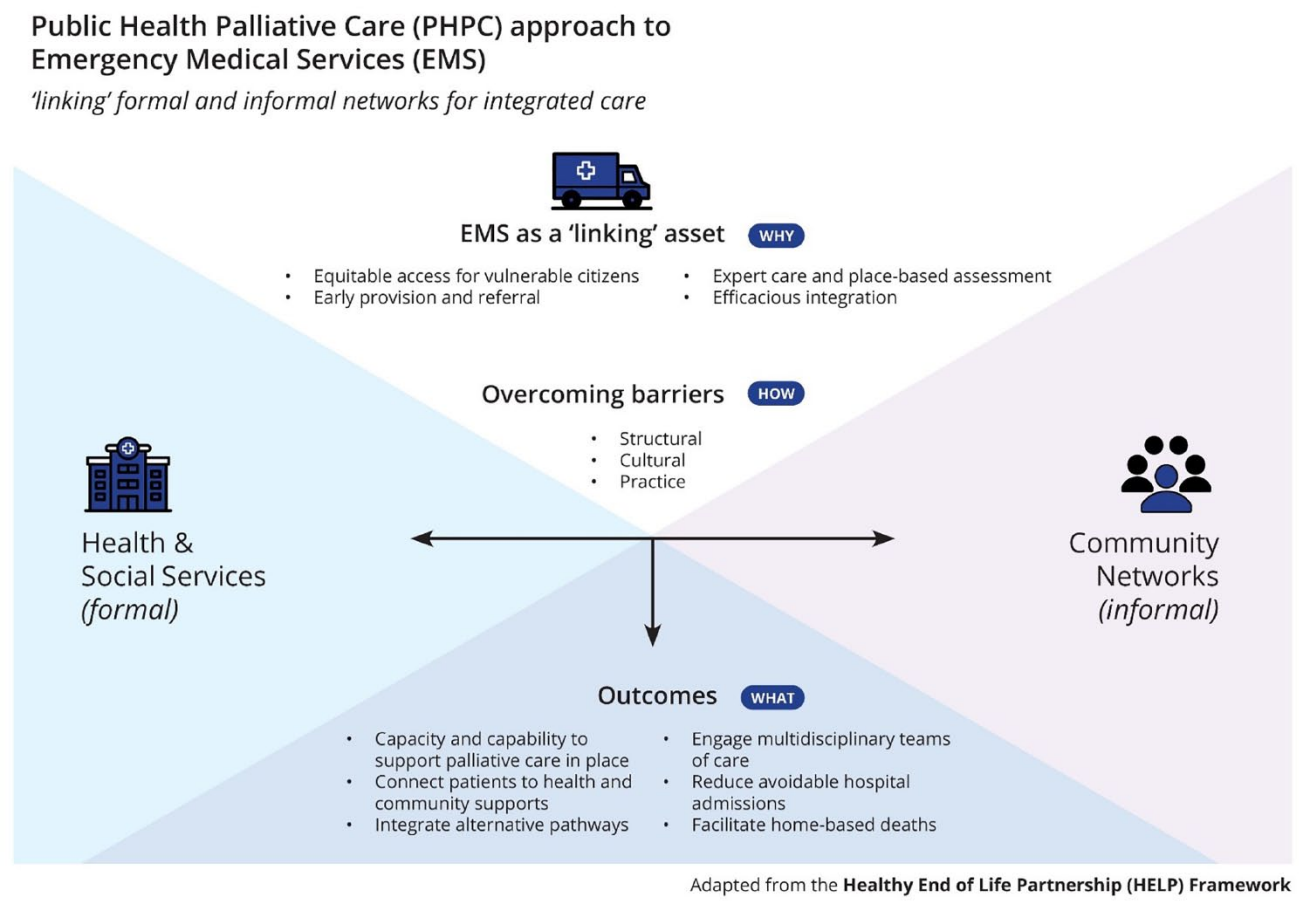
Public health palliative care approach to emergency medical services framework.

To address the barriers previously outlined, EMS ought to first be incorporated into national and international public health palliative care policies and frameworks. We have adapted the Healthy End of Life Program (HELP)^15,18^ partnership framework to develop a preliminary Public Health Palliative Care approach to Emergency Medical Services Framework (Framework) proposal for consideration (Figure 1).

This Framework aims to identify EMS as an asset capable of linking formal (health and social services) and informal (community) networks of palliative care to produce equitable access for marginalised and underserved groups in both HICs and LMICs; early provision and referral; expert care and place-based assessment; and efficacious integration. The Framework recognises that structural, cultural and practice barriers must be overcome, through the integration of health and social services with community networks, to produce desirable and sustainable outcomes. As a result, a public health palliative care approach to EMS could facilitate capacity and capability to support palliative care in place; connect patients to health and community supports; integrate alternative pathways; engage multidisciplinary teams of care; reduce avoidable hospital admissions; and facilitate home-based deaths.

To overcome the significant cultural barriers facing EMS in delivering palliative care, clinician attitudes and perceptions regarding the role of EMS must first evolve. Palliative care fundamentals ought to be imbued in education offerings to all EMS personnel, beginning at an undergraduate/student curriculum level and extending to ongoing mandated professional development training for established EMS personnel.^1^ Those exhibiting a particular aptitude or passion for palliative care need access to further opportunities to gain advanced practical training in palliative care with specialists, allowing these EMS personnel to become champions within their respective service and contribute to the ongoing sustainability of palliative care expertise.^70^

Patients, families and carers’ attitudes and perceptions of the potential function of EMS in community-based palliative care must also be addressed, given their important role in initiating EMS involvement in care with their call to emergency services. Previous studies that informed the development of the HELP framework highlighted:

Social attitudes to receiving palliative care help may be a barrier to building collaborative communities, which has profound implications for implementing public health approaches, such as compassionate communities. To be effective, any public health practice model must attempt to modify these social norms. The goals of a public health approach should be to create sustainable community environments with the capacity to engage in end of life discussion, support and practical care, whilst developing community norms that ensure citizens know about and can draw upon these assets when needed.^18^

Shifting societal attitudes to accept EMS as an asset of community-based palliative care, beyond their traditional hospital conveyance role, will require a targeted behaviour change campaign^71^ around death literacy and the role of EMS in palliative care provision. Death literacy is defined as ‘a set of knowledge and skills that make it possible to gain access to understand and act upon end-of-life and death care options’.^72^ EMS also have the unique opportunity to engage patients and their families and carers in palliative care health promotion, and sign-post locally provided health and community supports.^73^

Investing in the research and implementation of alternate referral pathways for EMS caring for palliative patients in the community must also be prioritised. Initiatives such as the PACER model could be adapted to a palliative care context; however, this would likely be resource intensive and only feasible in metropolitan settings of HICs. A pilot programme should first be conducted to investigate the efficacy of such a partnership, and pending evaluation success, this model of care could be implemented in suitable settings with adequate funding allowing for sustainable provision.

Other avenues to establish integrated models of community-based palliative care, harnessing the assets of health and social service workforces with community networks, must also be developed and piloted. The COVID-19 pandemic has highlighted significant opportunities for integrating specialist support into otherwise geographically inaccessible settings, and proven healthcare systems are adaptive to change.^74^ A recent study confirmed Australian EMS have a high level of intention to use a specialist palliative care telehealth service if it were made available to them.^75^

EMS chaplains can offer another avenue of integrated support for community-based palliative care patients and their families and carers. Chaplains can deliver reactive scene support to EMS personnel during palliative care call-outs, providing extended continuity of care and early bereavement to families and carers when the patient dies, allowing EMS to reduce time on scene and be dispatched to other patients.^76^

Future research could investigate key gaps in the current literature, including the differences in context between LMICs and HICs; underserved populations’ access and usage of palliative care EMS; and patient, family and carer perspectives.

Embedding a public health palliative care approach to EMS into health systems will ultimately facilitate community preferences to die in place and avoid hospitalisation where appropriate: a goal all countries, regardless of their SES, are likely aspiring towards.

## Conclusion

A public health approach to palliative and EoLC acknowledges and facilitates the contribution everyone can make in improving the experiences of seriously ill and dying people. The EMS sector, in particular, offers valuable resources and skills distinct from other services that positions them to provide expertise and support in situations that would otherwise go unmet. This article articulates a public health approach to EMS palliative and EoLC provision and offers a preliminary framework to illustrate the components of a potential implementation and policy strategy.

## Declarations

**Ethics approval and consent to participate:** Not applicable.

**Consent for publication:** Not applicable.

**Availability of data and materials:** Not applicable.
